# pH-controlled growth of triangular silver nanoprisms on a large scale

**DOI:** 10.1039/c9na00635d

**Published:** 2019-11-06

**Authors:** Zhishan Zhang, Ji Yu, Jianhui Zhang, Yadong Lian, Zeyu Shi, Zimo Cheng, Min Gu

**Affiliations:** National Laboratory of Solid State Microstructures, Department of Physics, Collaborative Innovation Center of Advanced Microstructures, Nanjing University Nanjing 210093 P. R. China mgu@nju.edu.cn zhangjh@nju.edu.cn

## Abstract

A simple, mild, and reproducible one-pot approach was developed to synthesize triangular silver nanoprisms (TSNPRs) on a large scale. The TSNPR size was tailored from 30 to 100 nm by varying the dosage of a sodium hydroxide pentanol solution in the water/polyvinylpyrrolidone/*n*-pentanol ternary system. The use of the sodium hydroxide pentanol solution modified the initial pH of the water/polyvinylpyrrolidone/*n*-pentanol system and made the synthesis of TSNPRs highly reproducible and independent of the polyvinylpyrrolidone pH. *N*,*N*-dimethyl formamide and formamide were used to control the system pH and improved the resultant TSNPRs in both syntheses repeatedly to have a well-defined shape. The extinction bands of the TSNPRs were relatively narrow, which makes them promising for chemical and biological applications.

## Abstract 2

A simple, mild, and reproducible one-pot approach was developed to synthesize triangular silver nanoprisms (TSNPRs) on a large scale.

## Graphical abstract

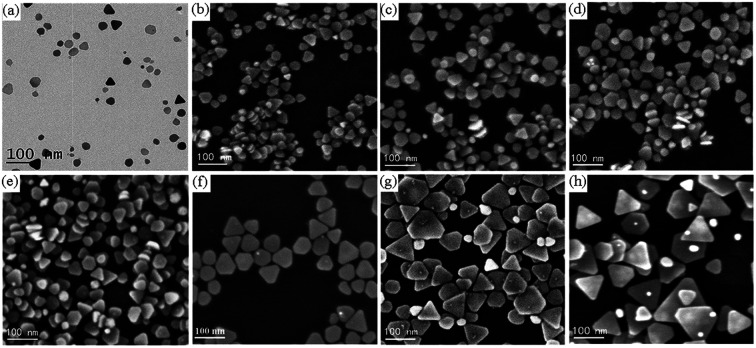

## Introduction

Over the recent decades, the shape-controlled synthesis of metallic nanoparticles has been of technological importance because of the strong correlation between the size, shape, and optical, electrical, chemical, and biological properties.^1–4^ Among metal nanoparticles, silver (Ag) nanoparticles are of particular interest. Because of their unique optical properties such as strong surface plasmon resonance and tunable light absorption by size, triangular silver nanoparticles (TSNPRs) have been used extensively in surface-enhanced Raman scattering, solar cells, conductive fillers in conductive adhesives and thermal interfacial materials, a class of broad-spectrum antimicrobial reagents, classic catalysts, biological detection,^5–14^*etc.* TSNPRs were first synthesized by Jin and coworkers using a plasmon-mediated method in 2001,^15^ and different strategies were used to prepare TSNPRs, including optical-induction methods,^5,16,17^ thermal transformation,^18–20^ seed-mediated methods,^21–24^ and template-directed growth.^25–27^ However, only a few of these methods can tune the Ag nanoprism size. The optical-induction methods can produce uniform TSNPRs, but their yields are poor. Furthermore, too many reagents are used in seed-mediated methods. Finally, complex synthesis procedures also limit these methods.

To simplify the synthesis route and to increase the TSNPR yield, we have developed a unique water/polyvinylpyrrolidone (PVP)/*n*-pentanol ternary system (WWPN) to prepare Ag nanoprisms.^28^ The WWPN system has provided simplified synthesis procedures and a significantly increased yield of about 20 mg. The resulting TSNPR is pure and its size can be tuned by adjusting the PVP amount. However, the pH of commercial PVP varies frequently, which is critical for our WWPN system. The pH of different batches of PVP (K30) varies from 3 to 5. When the pH of PVP is less than 3.6, the repeated synthesis of TSNPRs in the WWPN system usually fails. The initial pH of the WWPN system must be controlled to ensure the synthesis repeatability. By using the sodium hydroxide-pentanol solution (SHPS) innovatively, we controlled the initial pH of the WWPN system. The TSNPRs can be synthesized repeatedly independent of the PVP pH, and they can be tuned from 30 to 100 nm by adjusting the amount of the SHPS. To avoid the following precipitation reaction (1) between AgNO_3_ and NaOH,

1 2NaOH + 2AgNO_3_ = 2NaNO_3_ + Ag_2_O↓ + H_2_O

before adding AgNO_3_, the SHPS was added to the *n*-pentanol solution of PVP to increase the solution pH from 4 to 7. However, as indicated previously,^28^ most of the resultant TSNPRs were truncated. To improve the synthesis repeatability and triangular shape of the TSNPRs, *N*,*N*-dimethyl formamide (DMF) and formamide were used to control the system pH. DMF and formamide helped to synthesize TSNPRs repeatedly. The resultant TSNPRs had a well-defined triangular shape. By controlling the initial pH of the WWPN system, a simple, mild, and reproducible one-pot approach was developed to synthesize TSNPRs with a tunable size and excellent triangular shape on a large scale.

## Experimental

### Materials

Silver nitrate (AgNO_3_, AR, ≥99.8%), formamide (≥99.5%), DMF (≥99.5%), ethanol and sodium hydroxide (NaOH, AR, ≥96%) were purchased from Sinopharm Chemical Reagent Co. Ltd. *n*-Pentanol (≥99%) was obtained from Shanghai Lingfeng Chemical Reagent Co. Ltd. Polyvinylpyrrolidone (PVP, *M*_n_ = 30 000 g mol^−1^, pH = 3.64) was obtained from Shanghai Yeasen Biotechnology Co. Ltd. Water was distilled and deionized using a Millipore Milli-Q Purification System and has a resistivity of more than 18.2 MΩ cm. All reagents were used without further purification.

### Instruments

Extinction spectra of silver-nanoparticle dispersions were recorded using a UV-1800 spectrophotometer (Mapada). Scanning electron microscopy (SEM, FEI Helios 600i, operating voltage: 2 kV, operating current: 34 pA) was used to observe the morphology of the silver nanoparticles. X-ray diffraction (XRD) was performed on a Philips X'pert Pro using Cu-K_α1_ radiation (*λ* = 1.5406 Å) from 30 to 90°. Transmission electron microscopy (TEM) images were obtained using a JEM-2100 with an acceleration voltage of 200 kV. Samples for TEM analysis were prepared by adding a drop of the silver nanoparticle dispersion onto a copper grid and drying in air at room temperature.

### Synthesis of Ag nanoprisms by adding a SHPS

In a typical synthesis, 1.8 g of PVP was dissolved in 60 mL of *n*-pentanol in a conical flask sealed with a ground stopper. A certain amount of the SHPS (1 g L^−1^) was injected into the solution with stirring. After 30 min, 3.9 mL of aqueous AgNO_3_ (1.2 wt%) was added and stirred for 30 min to form the WWPN ternary system. The reaction solution in the sealed flask was placed in a constant-temperature oven and heated at 95 °C for about 42 h. The reaction products were washed with ethanol three times by centrifugation, and Ag nanoprisms were obtained.

### Synthesis of Ag nanoprisms by adding an organic base

0.6 g of PVP was dissolved in 60 mL of *n*-pentanol in a conical flask sealed with a ground stopper. 3.9 mL of aqueous AgNO_3_ (0.6 wt%) was added to the solution and stirred for 30 min. The reaction solution in the sealed flask was placed in a constant-temperature oven and heated at 95 °C for 12 h. 3 mL of DMF or formamide was added after the reaction solution was cooled to room temperature and stirred for 5 min. The reaction solution in the sealed flask was placed in a constant-temperature oven and heated at 95 °C for a further 50 h (for DMF) or 30 h (for formamide). The final products were washed with ethanol three times by centrifugation, and Ag nanoprisms were obtained.

## Results and discussion

### Synthesis of Ag nanoprisms by changing the pH

**Fig. 1:**
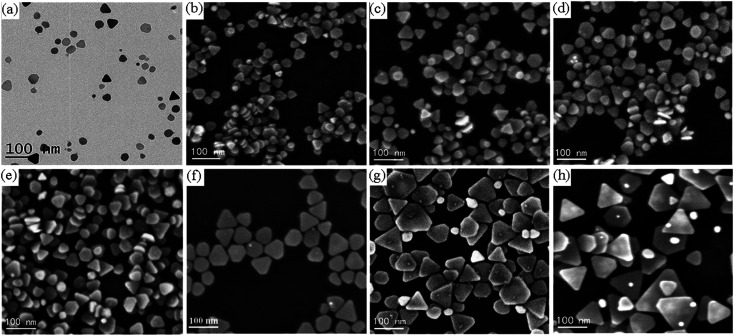
TEM and SEM images of Ag nanoprisms made with (a) 0.25 mL, (b) 0.375 mL, (c) 0.5 mL, (d) 0.75 mL, (e) 1.0 mL, (f) 1.25 mL, (g) 1.5 mL and (h) 2.0 mL of the SHPS. The average edge lengths of the corresponding Ag nanoprisms are (a) 30 ± 4 nm, (b) 35 ± 5 nm, (c) 45 ± 6 nm, (d) 50 ± 7 nm, (e) 60 ± 8 nm, (f) 70 ± 9 nm, (g) 80 ± 12 nm and (h) 100 ± 20 nm.

Fig. 1 shows typical TEM and SEM images of Ag nanoprisms prepared with different amounts of the SHPS. As shown in Fig. 1a–f, Ag nanoprisms with average edge lengths of 30 ± 4 nm, 35 ± 5 nm, 45 ± 6 nm, 50 ± 7 nm, 60 ± 8 nm, 70 ± 9 nm, 80 ± 12 nm and 100 ± 20 nm were synthesized by using 0.25 mL, 0.375 mL, 0.5 mL, 0.75 mL, 1 mL, 1.25 mL, 1.5 mL and 2 mL of the SHPS, respectively. However, when the SHPS was not added into the WWPN system, the synthesis of TSNPRs failed; when the amount of the SHPS was equal to or greater than 2.5 mL, a black precipitate was formed, indicating that silver oxide was precipitated. The influence of the SHPS amount on the nanoprism edge length is illustrated by linearly fitting using the following formula:

2 *y* = 21.47 + 39.12*x*

where *y* (nm) refers to the nanoprism edge length and *x* (mL) refers to the SHPS amount. The correlation coefficient is 0.997.

**Fig. 2:**
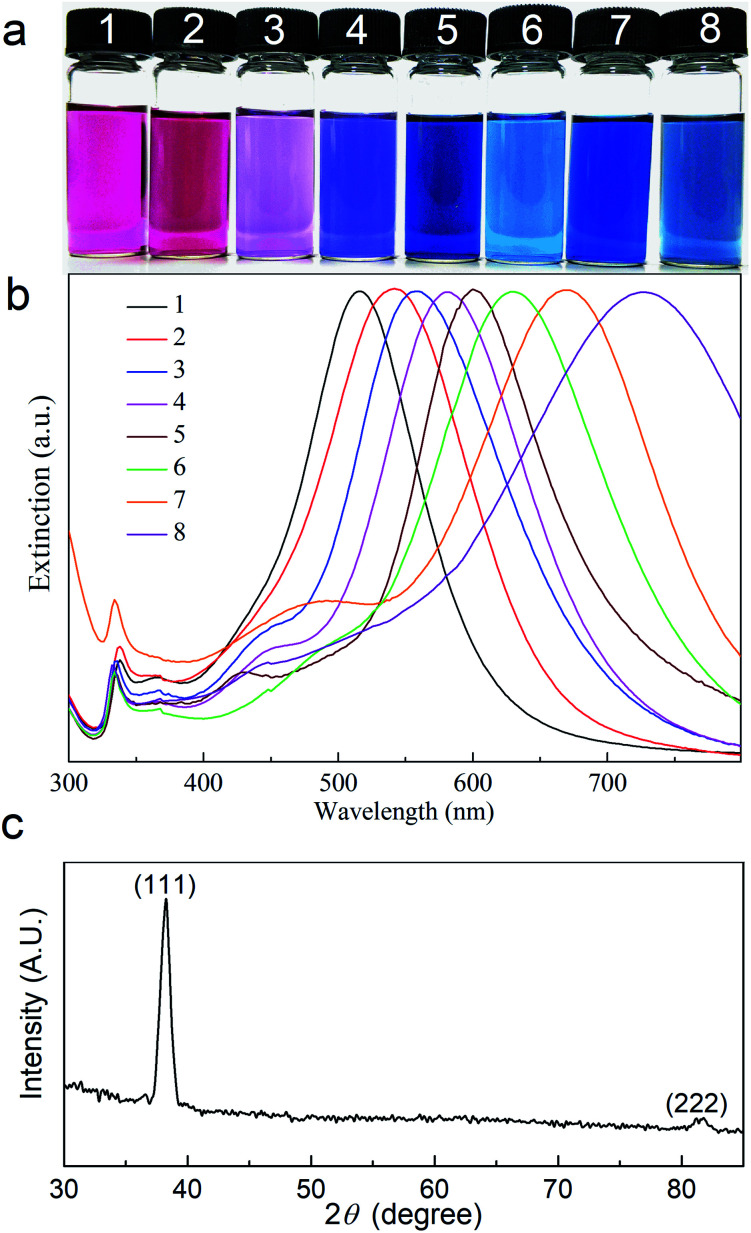
(a) Photographs and (b) extinction spectra for ethanol dispersions of Ag nanoprisms with average edge lengths of (1) 30 ± 4 nm, (2) 35 ± 5 nm, (3) 45 ± 6 nm, (4) 50 ± 7 nm, (5) 60 ± 8 nm, (6) 70 ± 9 nm, (7) 80 ± 12 nm and (8) 100 ± 20 nm, respectively. (c) XRD patterns of the Ag nanoprisms.

Photographs of ethanol dispersions of Ag nanoprisms with different average edge lengths are shown in Fig. 2a. These dispersions vary in color due to their LSPR properties from pink and purple to blue. The in-plane dipole plasmon resonance band of the Ag nanoprisms red-shifts with the increase of the average edge length of the Ag nanoprisms. Fig. 2b shows the extinction spectra for ethanol dispersions of Ag nanoprisms with different average edge lengths. Every spectral line has three distinctive peaks, a large strong peak at a long wavelength, a weaker broad peak, and a small sharp peak at a short wavelength. These peaks can be assigned to the in-plane dipole, in-plane quadrupole, and out-of-plane quadrupole plasmon resonance bands, respectively.^28^ As shown in Fig. 2b, the in-plane dipole plasmon-resonance peak blue-shifts to a short wavelength with an increase in the amount of the SHPS. The prominent (111) reflection of Ag nanoprisms at 2*θ* = 38.2° in Fig. 2c, shows that the (111) plane is the preferred orientation surface.

As shown in Fig. 1b–f, most Ag nanoprisms made with the SHPS were truncated. To obtain regular triangular Ag nanoprisms with sharp corners, DMF and formamide were investigated to improve the triangular shape of the Ag nanoprisms. DMF has been demonstrated as a solvent and reducing agent in the presence of PVP and has changed the preferential adsorption of PVP to {111} Ag facets.^29^ Pastoriza-Santos and coworkers proposed the mechanism outlined in formula (3):^20^

3 HCONMe_2_(DMF) + 2Ag^+^ + H_2_O → 2Ag^0^ + Me_2_NCOOH + 2H^+^

**Fig. 3:**
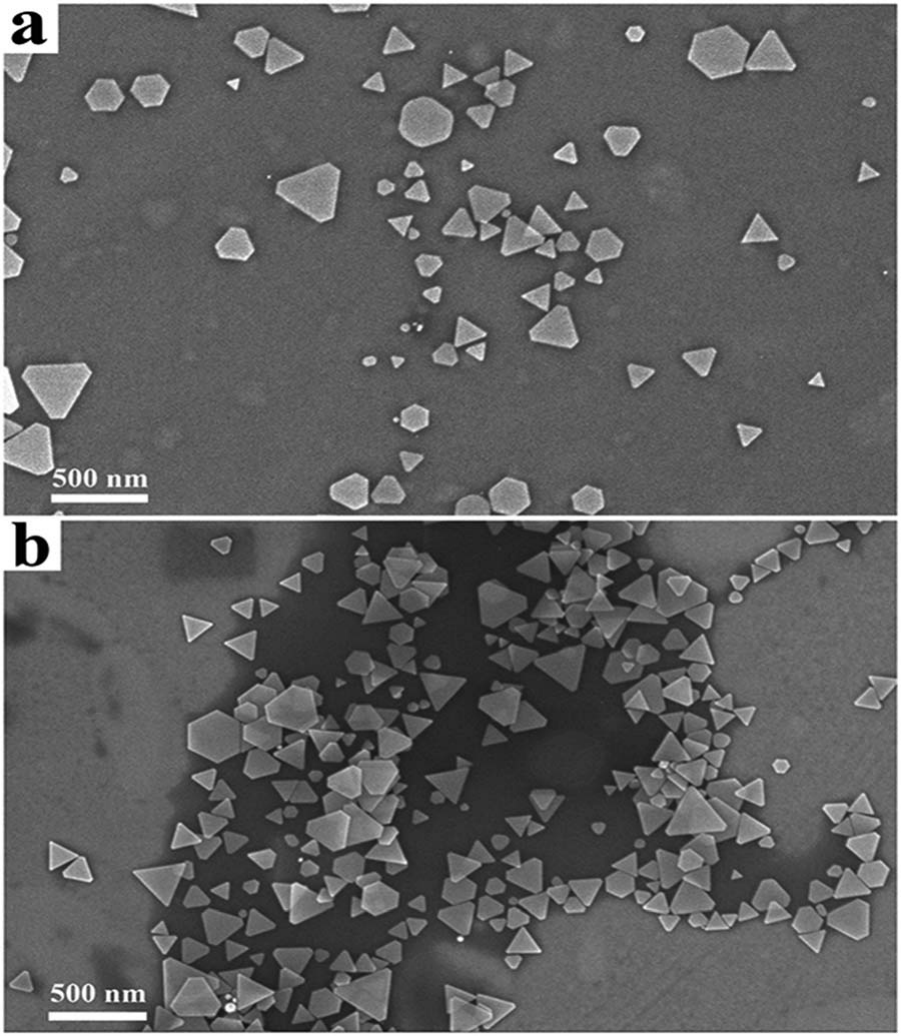
SEM images of Ag nanoprisms made with 0.6 g PVP and different annexing agents. (a) 3 mL DMF and (b) 3 mL formamide.

**Fig. 4:**
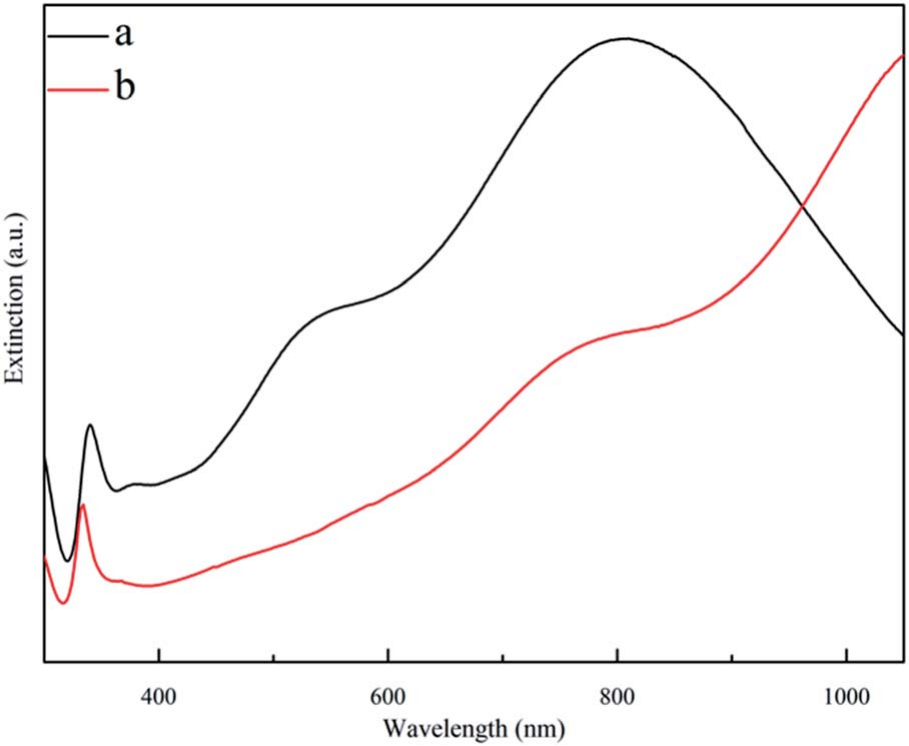
Extinction spectra for ethanol solutions of Ag nanoprisms made with 0.6 g PVP and different annexing agents. (a) 3 mL DMF and (b) 3 mL formamide.

As shown in Fig. 3a and 4a, the SEM images and extinction spectra show that the Ag nanoprisms made with DMF had a well-defined shape with sharp corners but an uneven size. As shown in Fig. 3b and 4b, the use of formamide improved the triangular shape of the Ag nanoprisms, but expanded the size-distribution range and led to broad extinction bands.

### Mechanism of the synthesis of Ag nanoprisms by changing the pH

Typically, the growth mechanisms of Ag nanoprisms can be divided into crystallographic and redox-chemistry arguments. Each methodology involves two general steps. One is nucleation of nanoprism seeds, and the other is crystal growth of seeds by a mediated reduction of metal ions.^30,31^ In the first step of the synthesis, when the reaction solution was heated, Ag^+^ ions diffused into the WWPN ternary system region and reacted with PVP by coordinating with O and N atoms of the pyrrolidone ring of PVP, and the Ag^+^–PVP complex was formed.^32^ Ag^+^–PVP was reduced to Ag^0^–PVP. The reactions may follow formulae (4) and (5).^33,34^4
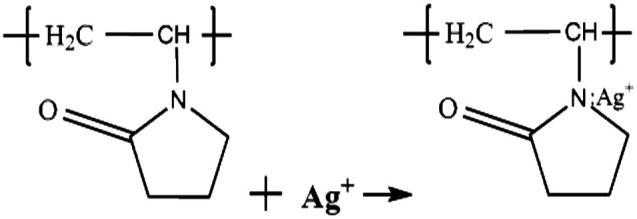
5
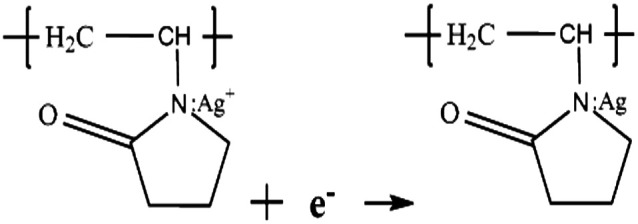


H^+^ ions formed ceaselessly as the reaction proceeded. An increased amount of H^+^ ions in the system depresses the reduction reaction and, ultimately, Ag nanoprism growth. Therefore, the pH of the reaction system can affect the reduction rate. When the pH of the reaction solution increased, the concentration of H^+^ ions in the reaction system decreased, and the reaction proceeded in the reduction direction, which promoted the growth of Ag nanoprisms. Therefore, as the amount of the SHPS increases, the size of the triangular Ag nanoprisms becomes larger. When the nucleation of nanoprism seeds and the crystal growth of seeds by the mediated reduction of metal ions reached a balance, Ag nanoprisms with a certain size were obtained. On the other hand, it is evident that the corners of the Ag nanoprisms were poorly truncated. This can be ascribed to the acidic environment caused by the H^+^ ion formation. Since H^+^ acts as an etchant in reaction, with the increasing of H^+^ concentration (pH decrease), the etching ability of the system becomes stronger, and the truncation reaction is more likely to occur.^35^ Thus, as seen in the SEM images, when the amount of the SHPS was equal to or less than 0.25 mL, truncated nanoprisms with round tips formed, and even some Ag nanospheres, rather than nanoprisms.

All the above experimental approaches were targeted towards controlling the initial pH of the reaction solution. The results proved that a higher initial pH led to the formation of Ag nanoprisms. Therefore, the pH controls the triangular Ag nanoprism growth.

The discussion above indicates that the Ag nanoprisms fabricated by adding the SHPS were size-tunable with a narrow size distribution but were truncated. The Ag nanoprisms made by adding an organic base had a well-defined shape with sharp corners but had a polydisperse size distribution. It is expected that the high-purity, uniform Ag nanoprisms with sharp corners can be obtained by combining the advantages of both methods mentioned above and reducing the shortcomings.

## Conclusions

By controlling the initial pH, the WWPN ternary system was developed to prepare TSNPRs reproducibly with a tunable size and a well-defined triangular shape on a large scale. The SHPS, DMF, and formamide were used to control the initial pH. Ag nanoprisms made with the SHPS were monodisperse and tunable in size, but their corners were usually truncated. The Ag nanoprisms made with DMF or formamide had a well-defined triangular shape, but they tended to be polydisperse.

## Conflicts of interest

There are no conflicts to declare.

## Supplementary Material
